# Supplementary material for: On the use of non-concurrent controls in platform trials: a scoping review
Source: Trials. 2023 Jun 15;24:408. doi: 10.1186/s13063-023-07398-7 (PMC10268466; doi:10.1186/s13063-023-07398-7)
Supplement: Supplementary file 1 — Additional file 1. In the first supplementary material, the process for the methods review, as well as the guideline review are described in detail. The list of pre-defined articles and the forms for the information extraction process are included. Besides, a list of the included articles and guidelines for extraction are presented. [file 13063_2023_7398_MOESM1_ESM.pdf]

Supplementary material of "On the use of non-concurrent controls in platform trials: A scoping review"

Marta Bofill Roig *[1], Cora Burgwinkel[1,2], Ursula Garczarek[3], Franz Koenig[1], Martin Posch[1], Quynh Nguyen[2], and Katharina Hees[2]

[1]*Section for Medical Statistics, Center for Medical Statistics, Informatics, and Intelligent Systems, Medical University of Vienna, Vienna*
[2]*Paul-Ehrlich Institut, Department of Biostatistics, Langen*
[3]*Cytel Inc., Strategic Consulting, Hagen*

In this supplementary material, the process for the methods review, as well as the guideline review are described in detail. The list of pre-defined articles and the forms for the information extraction process are also included.

1 Additional information regarding the methods review

In this section, we describe the protocol followed for the methods review, and present the extraction form used for the information extraction in the systematic review and list of included publications.

1.1 Protocol Methods review

A systematic search was carried out in the PubMed database and the identified articles were supplemented with manually searched papers. The following keywords were considered important and were used for the query: non-concurrent control(s), concurrent control(s), historical control(s), shared control(s), historical borrowing, external control(s). To identify the relevant methods that incorporate non-concurrent control data in PubMed, the following query was performed:

```
   ("non concurrent control*"[Title/Abstract] OR "control arm*"[Title]
   OR "concurrent control*"[Title] OR "historical control*"[Title]
   OR "external control*"[Title] OR "shared control*"[Title/Abstract])
AND
```

*marta.bofillroig@meduniwien.ac.at

```
  ("design*"[Title/Abstract] OR "stud*"[Title/Abstract]
  OR "platform trial*"[Title/Abstract]
  OR "master protocol*"[Title/Abstract])
AND
  ("trial*"[Title/Abstract] OR "clinical"[Title/Abstract])
```

Several searches which included, for example, the term "historical control*" in the abstract, resulted in such a large number of articles that it was infeasible to review. As result, a narrower search was performed (i.e. search term only included in the title) which prioritized the relevance of the articles in terms of methodology. Besides, it was of importance that the following a priori defined list of articles of interest was found by our search and that the most relevant articles reviewing historical control methods were also included:

- Including non-concurrent control patients in the analysis of platform trials: is it worth it? Lee and Wason (2020) [1]
- Beyond Randomized Clinical Trials: Use of External Controls, Schmidli et al. (2020) [2]
- Utilizing shared internal control arms and historical information in small-sized platform clinical trials, Jiao et al. (2019) [3]
- Robust meta-analytic-predictive priors in clinical trials with historical control information, Schmidli et al. (2014) [4]
- Use of historical control data for assessing treatment effects in clinical trials, Viele et al. (2014) [5]
- Incorporating historical control data in planning phase II clinical trials, Thall and Simon (1990) [6]

This study is reported in accordance with the PRISMA reporting guideline's extension for scoping reviews [7]. The query led to 260 articles (date of search 17/08/2021) and the articles were included if one of the following inclusion criteria was fulfilled:
1) The description of a method proposed to include external/non-concurrent controls and concurrent controls in clinical trials.
2) The article considered an application of a method which included external/ non-concurrent controls together with concurrent controls in a clinical trial context with a detailed description of the method used.
3) The article is an overview of several methods to include external controls (e.g. review article).
4) The article is about the comparison of several methods (e.g. via simulation studies) to include external controls.

The article was not considered if:
1) The article is a duplicate.

2) The article focuses on shared controls but not on the inclusion of external controls.
3) The term external control is used in another context.
4) The article focuses on a clinical trial using external controls, but not on the methods.

The screening of the articles was performed by two reviewers. In the first step, article titles were screened by a reviewer and selected based on the inclusion and exclusion criteria above. Then, the article abstracts were screened and selected by a reviewer based on the same inclusion and exclusion criteria. In the next step, the selected articles were fully read and screened by two reviewers based on the inclusion criteria and exclusion criteria.

Regarding the information extraction process, two independent reviewers read the identified articles and extracted the information using a standardized data extraction form (see Table 1). Where possible, pre-specified categories were defined for each item in the extraction form. As one can see in Table 1, information about the publication such as year and journal of publication, objective of article and type of article (e.g. review, discussion, research article) were extracted. Besides, information concerning the study design, such as type of control and type of endpoints were identified. Regarding the incorporation of external/internal controls, details on the statistical methodology, such as covariate adjustment and interim analyses, were collected. Further information was gathered concerning the implementation of simulation and/or case studies and the availability of code and software. After the information extraction, a third person, the adjudicator, compared the extracted information and checked for discrepancies. In the end, the number of articles in each pre-specified category was determined and free-text fields were summarized in listings.

Following the selection steps above, 44 articles were included for the full text review. Further 11 articles were manually added (see list above), such that in total 55 articles were included in the full text review. During the full text review, 12 articles were excluded resulting in 43 articles for the information extraction process (for a full list of included articles see Table 2).

Table 1: Extraction form for systematic review

Question	Possible options or [Data type]
**Publication**	
1. Title of article	[Text]
2. First author	[Text]
3. Year of Publication	[Integer]
4. DOI	[Integer]
5. Journal of publication	[Text]
6. Objective of article	[Text]
7. Type of article	A/ B/ C [A: Review; B: Discussion; C: Research Article]
**Study design**	
8. Is the article focused on clinical trials? If yes	Yes/ No
8a. Does it focus on platform studies?	Yes/ No
9. Population in the concurrent/ actual trial	A/ B/ C [A: Several populations are included; B: Only one population is included; C: Not specified]
10. Type of control	A/ B [A: External controls; B: Internal controls]
11. Number of treatment arms (control arm not included)	A/ B/ C/ D [A: 1 arm; B: 2 arms; C: +2 arms; D: Not specified]
12. Type of endpoint(s) considered	A/ B/ C/ D/ E [A: Binary endpoint; B: Continuous endpoint; C: Survival endpoint; D: Others; E: Not specified]
**Statistical methodology**	
13. Method(s) used for incorporating non-current controls	[Text]
14. Type of method to incorporate non-concurrent controls	A/ B/ C [A: Frequentist approach; B: Bayesian approach; C: Hybrid approach]
15. Is the method downweighting the non-concurrent controls depending on the differences between concurrent and non-concurrent controls?	Yes/ No
16. Modelling-based approach: Is the method modelling time? If yes,	Yes/ No
16a. Does it assume any distribution of time trends?	Yes/ No
16b. How are time trends incorporated into the model/ method?	[Text]
17. Can the method adjust for covariates?	A/ B/ C [A: Yes; B: No; C: Not specified]
18. Was the potential bias discussed?	Yes/ No
19. Are interim analyses also covered?	Yes/ No
**Simulation/ Case Studies**	
20. Were any simulations or case studies covered? If yes,	Yes/ No
21. What study design was used for the simulations (e.g. allocation ratio, treatment arms, type of endpoint)?	[Text]
22. Was the data-generating model described? If yes,	Yes/ No
22a. Which distribution was used to simulate the responses?	[Text]
23. Were the simulations based on a real dataset? If yes,	Yes/ No
23a. Was a real data set used to decide on the parameter values?	Yes/ No
24. How was the performance of the method evaluated?	[Text]
25. Were scenarios under the null hypothesis considered?	Yes/ No
26. Were scenarios under the alternative hypothesis considered?	Yes/ No
27. Were time trend patterns simulated? If yes,	Yes/ No
27a. Which time trend patterns were used?	[Text]
28. Were the simulations performed to compare the methods to another?	Yes/ No
**Software**	
29. What package and/ or software was used?	[Text]
30. Is the software or code available?	Yes/ No
**Limitations and Conclusion**	
31. What are limitations of the study and what is the general conclusion?	[Text]

Table 2: List of included publications

Title	First author	Journal
Minimizing control group allocation in randomized trials using dynamic borrowing of external control data - An application to second line therapy for non-small cell lung cancer	Dron L	Contemp Clin Trials
Utilizing shared internal control arms and historical information in small-sized platform clinical trials	Jiao F	J Biopharm Stat
Borrowing from Historical Control Data in Cancer Drug Development: A Cautionary Tale and Practical Guidelines	Lewis CJ	Stat Biopharm Res
A Comparison Between a Meta-analytic Approach and Power Prior Approach to Using Historical Control Information in Clinical Trials With Binary Endpoints	Isogawa N	Ther Innov Regul Sci
Incorporating individual historical controls and aggregate treatment effect estimates into a Bayesian survival trial: a simulation study	Brard C	BMC Med Res Methodol
Design of randomized controlled confirmatory trials using historical control data to augment sample size for concurrent controls	Yuan J	J Biopharm Stat
A practical Bayesian adaptive design incorporating data from historical controls	Psioda MA	Stat Med
Bayesian selective response-adaptive design using the historical control	Kim MO	Stat Med
Use of a historical control group in a noninferiority trial assessing a new antibacterial treatment: A case study and discussion of practical implementation aspects	Dejardin D	Pharm Stat
Covariate-adjusted borrowing of historical control data in randomized clinical trials	Han B	Pharm Stat
Robust meta-analytic-predictive priors in clinical trials with historical control information	Schmidli H	Biometrics
Use of historical control data for assessing treatment effects in clinical trials	Viele K	Pharm Stat
Using historical control information for the design and analysis of clinical trials with overdispersed count data	Gsteiger S	Stat Med
Adaptive adjustment of the randomization ratio using historical control data	Hobbs BP	Clin Trials
The inclusion of historical control data may reduce the power of a confirmatory study	Cuffe RL.	Stat Med
Incorporating historical control data in planning phase II clinical trials	Thall PF	Stat Med
Statistical considerations of phase 3 umbrella trials allowing adding one treatment arm mid-trial	Ren Y	Contemp Clin Trials
The Use of External Control Data for Predictions and Futility Interim Analyses in Clinical Trials	Ventz S	Neuro-Oncology
The Use of External Controls in FDA Regulatory Decision Making	Jahanshahi M	Ther Innov Regul Sci
The use of external controls: To what extent can it concurrently be recommended?	Burger HU	Pharm Stat
Bayesian semiparametric meta-analytic-predictive prior for historical control borrowing in clinical trials	Hupf B	Stat Med
A novel equivalence probability weighted power prior for using historical control data in an adaptive clinical trial design: A comparison to standard methods	Bennett M	Pharm Stat
A roadmap to using historical controls in clinical trials - by Drug Information Association Adaptive Design Scientific Working Group (DIA-ADSWG)	Ghadessi M	Orphanet J Rare Dis
Historical Controls in Randomized Clinical Trials: Opportunities and Challenges	Hall KT	Clin Pharmacol Ther
Including non-concurrent control patients in the analysis of platform trials: is it worth it?	Lee KM	BMC Med Res Methodol
Reducing Patient Burden in Clinical Trials Through the Use of Historical Controls: Appropriate Selection of Historical Data to Minimize Risk of Bias	Lim J	Ther Innov Regul Sci
Propensity score-integrated composite likelihood approach for augmenting the control arm of a randomized controlled trial by incorporating real-world data	Chen WC	J Biopharm Stat
Critical appraisal of Bayesian dynamic borrowing from an imperfectly commensurate historical control	Harun N	Pharm Stat
An efficient Bayesian platform trial design for borrowing adaptively from historical control data in lymphoma	Normington J	Contemp Clin Trials
Beyond Randomized Clinical Trials: Use of External Controls	Schimidli H	Clin Pharmacol Ther
Clustered allocation as a way of understanding historical controls: Components of variation and regulatory considerations	Collignon O	Stat Methods Med Res
Bayesian leveraging of historical control data for a clinical trial with time-to-event endpoint	Roychoudhury S	Stat Med
A note on the power prior	Neuenschwander B	Stat Med
Power Prior Distributions for Regression Models	Ibrahim JG	Stat Sci
Modified power prior with multiple historical trials for binary endpoints	Banbeta A	Stat Med
Elastic priors to dynamically borrow information from historical data in clinical trials	Jiang L	Biometrics
The combination of randomized and historical controls in clinical trials	Pocock	J Chron Dis
Hierarchical Commensurate and Power Prior Models for Adaptive Incorporation of Historical Information in Clinical Trials	Hobbs BP	Biometrics
Elastic meta-analytic-predictive prior for dynamically borrowing information from historical data with application to biosimilar clinical trials	Zhang W	Contemp Clin Trials
Summarizing historical information on controls in clinical trials	Neuenschwander B	Clin Trials
A Bayesian model with application for adaptive platform trials having temporal changes	Wang C	Biometrics
The Bayesian Time Machine: Accounting for Temporal Drift in Multi-arm Platform Trials	Saville B. R.	Clin Trials
Adaptive power priors with empirical Bayes for clinical trials	Gravestock	Pharm Stat

2 Additional information regarding the guideline review

Next, we describe the protocol followed for the guideline review. We also present the extraction form for this review and list the reviewed guidelines.

2.1 Protocol for the guideline review

This systematic review was performed in accordance with the PRISMA reporting guideline's extension for scoping reviews [7]. The database for our systematic review of guidelines was based on all guidelines available for download on 20/05/2021. We accessed all scientific guidelines from the database of the European Medicine Agency[1] (EMA) with the following filters

- "Topic=Scientific guidelines",
- "Categories=Human",
- "Type of content=Documents"and
- "Include Documents=Yes"

while we used the following filters for the database of the U.S. Food and Drug Administration[2] (FDA)

- "Product=Drugs" and
- "Product=Biologics"

Then, we used the advanced search function of Adobe Acrobat Pro 2020 to search in all the PDF documents for the following keywords:

```
non-concurrent control(s)
concurrent control(s)
historical control(s)
shared control(s)
historical borrowing
external control(s)
master protocol(s)
bayesian method(s)
```

[1]https://www.ema.europa.eu/en/search/search

[2]https://www.fda.gov/regulatory-information/search-fda-guidance-document

Duplicates and older draft versions were excluded after the keyword search. Afterwards, we screened the identified documents and included

- guideline documents,
- Questions and Answers (QnAs),
- qualification opinion and
- reflection papers

from EMA, ICH or FDA for text extraction. We excluded documents

- in which external/non-concurrent controls were not discussed in the context of an inclusion into the primary analysis (e.g. the use of external/non-concurrent controls were just mentioned in the context of sample size planning),
- in which one of the keywords and hence, the use of external/non-concurrent controls was only mentioned without further recommendation or description (e.g. mentioned only in the title of a reference)
- in which the use of external/non-concurrent controls was only mentioned in a non-clinical or preclinical setting
- or in the context of (secondary) safety data analyses or meta-analyses
- in which the use of external/non-concurrent controls was discussed in a medical device context.

Following the inclusion and exclusion of documents, two independent reviewers used a standardized data extraction form (see Table 3) to extract the relevant information. Where possible, pre-specified categories were defined for each item in the extraction form. General information of the guidelines such as the year of the guideline or the type of document (e.g. guideline, reflection paper) were extracted. We further documented whether the guideline discussed the use of external/non-concurrent data in an early or late phase and whether the guideline was focused on methodological or clinical aspects. Concerning the use of external/non-concurrent controls, details on the specific circumstances were extracted in which the use was recommended or deemed acceptable, or unacceptable, the concerns that were raised as well as the requirements for the use. Furthermore, the methods mentioned for the incorporation of external/non-concurrent data and the type of inferential question addressed were collected. We specifically identified whether the use of non-concurrent controls, or the joint use of external and concurrent controls in platform trials was discussed in the guideline. Adjudication was performed by a third reviewer in case of discrepancies.

After the text extraction, the number of guidelines in each pre-specified category was determined and free-text fields were summarized in listings.

Overall, 1527 documents were downloaded from the EMA and FDA database (download date: 20/05/2021): 176 documents from the EMA database and 1351 documents from the FDA website. After searching all the documents for the above keywords, 232 documents were identified. In total, 97 documents were included for the screening after exclusion of duplicates and old drafts. Based on the above inclusion and exclusion criteria, 60 guidelines were excluded, resulting in 37 guidelines for the final review and data extraction (for a full list of included guidelines see Table 4).

Table 3: Extraction form for guideline review

Question	Possible options or [Data type]
Title of the document	[Text]
1. Year of the document	[integer] [Note: if unknown, leave blank]
2. Type of document	Guideline/Reflection paper/QnA/Other
3. Source of the guideline	FDA/ICH/EMA
4. Does the document focus on the use of historical/external control in early or late phase trials?	Early/Late/Both/Not mentioned
5. General methodological or clinical indication specific document?	Methodological/Clinical/Both/Unclear
6. Use of external controls under specific circumstances recommended or deemed acceptable?	3/2/1/0 [ordinal: 3=Recommended; 2= Not recommended but acceptable; 1= Not acceptable, 0=Unclear]
7. If yes, what are the specific circumstances mentioned in which the use might be acceptable	[Note: if not recommended in 6, leave subquestions blank, "No" here means, that the circumstance is not specifically mentioned in the guideline]
Rare disease/event	Yes/No
Pediatric	Yes/No
Unmet medical need	Yes/No
High mortality	Yes/No
Long treatment period before endpoint can be measured	Yes/No
Indication specific	Yes/No
If indication specific yes, which indication:	[Text] [Note: if unknown, leave blank]
Large treatment effect	Yes/No
No time trend in disease population/management	Yes/No
Homogenous treatment effect	Yes/No
Ethical concerns regarding assignment to placebo/control	Yes/No
Predictable disease course/Natural history well defined	Yes/No
Predictable mortality	Yes/No
Objective endpoint	Yes/No
Drug effect self-evident	Yes/No
Feasibility of randomized trial	Yes/No
Other	[Text] [Note: if unknown, leave blank]
8. Concerns and/or requirements raised with the use of non-concurrent control data?	
Bias	Yes/No
Comparability	Yes/No
Data/Trial integrity	Yes/No
Indication specific concern or requirement	Yes/No
If indication specific yes, which indication specific concern:	[Text] [Note: if unknown, leave blank]
Yes, but no further details	Yes/No
Differences in measurements	Yes/No
Change in SOC	Yes/No
Selection bias	Yes/No
High quality of data	Yes/No
Other	[Text] [Note: if unknown, leave blank]
9. Methods mentioned for the incorporation	
Matching approach	Yes/No
Bayesian method (Power Prior, MAP, â€¦)	Yes/No
Meta-analysis	Yes/No
Full pooling	Yes/No
Threshold crossing, boundary for hypothesis testing	Yes/No
Modelling approach (e.g. regression model)	Yes/No
Other	[Text] [Note: if unknown, leave blank]
10. Use of Bayesian methods supported?	2/1/0 [ordinal: 2=Discussed and supported; 1=Discussed and not supported; 0=Not discussed]
11. Type of inferential question addressed in guideline:	
Non-inferiority	Yes/No
Superiority	Yes/No
Equivalence	Yes/No
not specified	Yes/No
12. Is the use of non-concurrent controls platform trials discussed?	Yes/No
13. Is specifically the joint use of external and concurrent controls discussed?	Yes/No

Table 4: List of included guidelines

Guideline	Year	Source
ICH guideline E8 (R1) on general considerations for clinical studies	2019	ICH
Guidance for Industry -E 10 Choice of Control Group and Related Issues in Clinical Trials	2001	ICH
Guidance for Industry - E11(R1) Addendum: Clinical Investigation of Medicinal Products in the Pediatric Population	2018	ICH
Guidance for Industry - Neglected Tropical Diseases of the Developing World: Developing Drugs for Treatment or Prevention	2014	FDA
Guidance for Industry - Non-Inferiority Clinical Trials to Establish Effectiveness	2016	FDA
Inborn Errors of Metabolism That Use Dietary Management: Considerations for Optimizing and Standardizing Diet in Clinical Trials for Drug Product Development	2018	FDA
Guidance for Industry - Rare Diseases: Common Issues in Drug Development - Draft Guidance	2019	FDA
Demonstrating Substantial Evidence of Effectiveness for Human Drug and Biological Products	2019	FDA
Interacting with the FDA on Complex Innovative Trial Designs for Drugs and Biological Products	2020	FDA
GUIDELINE FOR THE FORMAT AND CONTENT OF THE CLINICAL AND STATISTICAL SECTIONS OF AN APPLICATION	1988	FDA
ICH Topic E3 Structure and Content of Clinical Study Reports	1996	ICH
Guidance for Industry - Influenza: Developing Drugs for Treatment and/or Prophylaxis	2011	FDA
Guidance for Industry - Time and Extent Applications for Nonprescription Drug Products	2011	FDA
Guidance for Industry - Expedited Programs for Serious Conditions – Drugs and Biologics	2014	FDA
Guidance for Industry - Chronic Hepatitis C Virus Infection: Developing Direct-Acting Antiviral Drugs for Treatment	2017	FDA
Amyotrophic Lateral Sclerosis: Developing Drugs for Treatment	2019	FDA
Acute Myeloid Leukemia: Developing Drugs and Biological Products for Treatment	2020	FDA
Guidance for Industry - COVID-19: Master Protocols Evaluating Drugs and Biological Products for Treatment or Prevention	2021	FDA
Postapproval Pregnancy Safety Studies	2019	FDA
Guidance for Industry -Antibacterial Therapies for Patients With an Unmet Medical Need for the Treatment of Serious Bacterial Diseases	2017	FDA
Guidance for Industry - Duchenne Muscular Dystrophy and Related Dystrophinopathies: Developing Drugs for Treatment	2018	FDA
Guidance for Industry -Clinical Trial Endpoints for the Approval of Cancer Drugs and Biologics	2018	FDA
Adaptive Designs for Clinical Trials of Drugs and Biologics	2019	FDA
Reflection paper on the use of extrapolation in the development of medicines for paediatrics	2018	EMA
Guideline on quality, non-clinical and clinical requirements for investigational advanced therapy medicinal products in clinical trials	2019	EMA
GUIDELINE ON THE EVALUATION OF ANTICANCER MEDICINAL PRODUCTS IN MAN	2019	EMA
Guideline on the evaluation of medicinal products indicated for treatment of bacterial infections	2011	EMA
Reflection paper on the regulatory requirements for vaccines intended to provide protection against variant strain(s) of SARS-CoV-2	2021	EMA
Qualification opinion on Cellular therapy module of the European Society for Blood & Marrow Transplantation (EBMT) Registry	2019	EMA
Expedited Programs for Regenerative Medicine Therapies for Serious Conditions	2019	FDA
Human Gene Therapy for Neurodegenerative Diseases	2021	FDA
Safety, Efficacy, and Pharmacokinetic Studies to Support Marketing of Immune Globulin Intravenous (Human) as Replacement Therapy for Primary Humoral Immunodeficiency	2008	FDA
Considerations for Allogeneic Pancreatic Islet Cell Products	2009	FDA
Preparation of IDEs and INDs for Products Intended to Repair or Replace Knee Cartilage	2011	FDA
Clinical Considerations for Therapeutic Cancer Vaccines	2011	FDA
Human Gene Therapy for Rare Diseases	2020	FDA
FDARA Implementation Guidance for Pediatric Studies of Molecularly Targeted Oncology Drugs: Amendments to Sec. 505B of the FD&C Act	2021	FDA

References

[1] Kim May Lee and James Wason. Including non-concurrent control patients in the analysis of platform trials: Is it worth it? *BMC Medical Research Methodology*, 20(1):1–12, 2020. ISSN 14712288. doi: 10.1186/s12874-020-01043-6.

[2] Heinz Schmidli, Dieter A. Häring, Marius Thomas, Adrian Cassidy, Sebastian Weber, and Frank Bretz. Beyond randomized clinical trials: Use of external controls. *Clinical pharmacology & therapeutics*, 107(4):806–816, 2020. ISSN 0009-9236. doi: 10.1002/cpt.1723.

[3] Feiran Jiao, Wenda Tu, Sara Jimenez, Victor Crentsil, and Yeh Fong Chen. Utilizing shared internal control arms and historical information in small-sized platform clinical trials. *Journal of Biopharmaceutical Statistics*, 29(5):845–859, 2019. ISSN 15205711. doi: 10.1080/10543406.2019.1657132. URL https://doi.org/10.1080/10543406.2019.1657132.

[4] Heinz Schmidli, Sandro Gsteiger, Satrajit Roychoudhury, Anthony O Hagan, David Spiegelhalter, and Beat Neuenschwander. Robust Meta-Analytic-Predictive Priors in Clinical Trials with Historical Control Information. *Biometrics*, (December):1023–1032, 2014. doi: 10.1111/biom.12242.

[5] Kert Viele, Scott Berry, Beat Neuenschwander, Billy Amzal, Fang Chen, Nathan Enas, Brian Hobbs, Joseph G. Ibrahim, Nelson Kinnersley, Stacy Lindborg, Sandrine Micallef, Satrajit Roychoudhury, and Laura Thompson. Use of historical control data for assessing treatment effects in clinical trials. *Pharmaceutical statistics*, 13(1):41–54, 2014. ISSN 15391612. doi: 10.1002/pst.1589.

[6] Peter F. Thall and Richard Simon. Incorporating historical control data in planning phase ii clinical trials. *Statistics in medicine*, 1990. doi: 10.1002/sim.4780090304.

[7] Matthew J Page, Joanne E McKenzie, Patrick M Bossuyt, Isabelle Boutron, Tammy C Hoffmann, Cynthia D Mulrow, Larissa Shamseer, Jennifer M Tetzlaff, Elie A Akl, Sue E Brennan, Roger Chou, Julie Glanville, Jeremy M Grimshaw, Asbjørn Hróbjartsson, Manoj M Lalu, Tianjing Li, Elizabeth W Loder, Evan Mayo-Wilson, Steve McDonald, Luke A McGuinness, Lesley A Stewart, James Thomas, Andrea C Tricco, Vivian A Welch, Penny Whiting, and David Moher. The prisma 2020 statement: an updated guideline for reporting systematic reviews. *BMJ*, 372, 2021. doi: 10.1136/bmj.n71. URL https://www.bmj.com/content/372/bmj.n71.
